# Torsade de Pointes Due to Hypokalemia and Hypomagnesemia

**DOI:** 10.21980/J8JP8G

**Published:** 2022-10-15

**Authors:** Mary Crista Cabahug, Amrita Vempati

**Affiliations:** * Creighton University School of Medicine Phoenix Program, Valleywise Health Medical Center, Department of Emergency Medicine, Phoenix, AZ

## Abstract

**Audience:**

This scenario was developed to educate emergency medicine (EM) interns but can be used to educate medical students and junior residents.

**Introduction:**

Torsade de Pointes (TdP) is a rare but potentially fatal arrythmia if not quickly diagnosed and properly treated. TdP is defined as a polymorphic ventricular tachycardia (VT) characterized by an oscillatory change in amplitude around an isoelectric line that is associated with a QTc prolongation on the electrocardiogram (ECG).^1^ It has been well described to predispose to ventricular fibrillation and arrhythmic death. QTc prolongation can be congenital or acquired. Between 1 in 2000 to 20,000 have the genetic mutation for QTc prolongation.^1^ Acquired QTc is most commonly drug related leading to electrolyte abnormalities. 2 Around 28% of cases of TdP are associated with hypokalemia and hypomagnesemia.^2^ Several European centers estimate 0.8 to 1.2 per million people per year are drug induced.^1^ Patients with TdP most commonly presents with syncope, palpitations, and dizziness.^2^ While 50% are asymptomatic, up to 10% of patients will present in cardiac arrest.^1^ It is imperative for EM physicians to be able to recognize TdP as it can quickly decompensate into a ventricular fibrillation and sudden death. These patients require management of electrolyte abnormalities, ventricular dysrhythmias, and cardiac death.^2^ This simulation case will demonstrate treatment strategies for TdP with electrolyte repletion, antiarrhythmics, and defibrillation.

**Educational Objectives:**

By the end of this simulation session, learners will be able to: 1) formulate appropriate work-up for altered mental status (AMS) 2) recognize hypokalemia and associated findings on ECG 3) address hypomagnesemia in a setting to hypokalemia 4) manage pulseless VT by following advanced cardiac life support (ACLS) 5) recognize and address TdP 6) provide care after return of spontaneous circulation (ROSC) 7) consult intensivist and admit to intensive care unit (ICU).

**Educational Methods:**

This session was conducted using high-fidelity simulation, which was immediately followed by an in-depth debriefing session. Each session had three EM first-year residents and six observers. There was one simulation instructor running the session and one simulation technician who acted as a nurse.

**Research Methods:**

After the simulation and debriefing session was complete, an online survey was sent via surveymonkey.com to all the participants. The survey collected responses to the following questions: (1) was the case believable? (2) did the case have the right amount of complexity? (3) did the case help improve medical knowledge and patient care? (4) did the simulation environment gave a real-life experience? (5) did the debriefing session after simulation help improve knowledge? A Likert scale was used to collect the responses.

**Results:**

This case was performed once a year for 2 years in a row. There was a total of 19 respondents from both years. One hundred percent of them either agreed or strongly agreed that the case was beneficial in learning and in improving medical knowledge and patient care. All of them found the post-session debrief to be very helpful. Two of them felt neutral about the case being realistic.

**Discussion:**

This high-fidelity simulation was a realistic way of educating learners on how to manage hypokalemia and hypomagnesemia leading to TdP. Cost-effectiveness varies depending on what is available at individual simulation laboratories. Learners are forced to start with a broad differential for the patient who presents with AMS. As they manage the case, the patient quickly decompensates into a fatal arrhythmia due to electrolyte abnormalities. Learners enforced their knowledge on leading ACLS, intubation skills, and treating TdP with electrical conversion and electrolyte repletion.

**Topics:**

Hypokalemia, hypomagnesemia, torsades de pointes, altered mental status, medical simulation.

## USER GUIDE

**Table:** 

**List of Resources:**	
Abstract	27
User Guide	29
Instructor Materials	31
Operator Materials	42
Debriefing and Evaluation Pearls	44
Simulation Assessment	47

**Learner Audience:**

Medical Students, Interns, Junior Residents

**Time Required for Implementation:**

Instructor Preparation: 20–30 minutes

Time for case: 15–20 minutes

Time for debriefing: 30–40 minutes

**Recommended Number of Learners per Instructor:**

3

**Topics:**

Hypokalemia, hypomagnesemia, torsades de pointes, altered mental status, medical simulation

**Objectives:**

By the end of this simulation session, learners will be able to:

1. Formulate appropriate work-up for altered mental status (AMS)
2. Recognize hypokalemia and associated findings on ECG
3. Address hypomagnesemia in a setting of hypokalemia
4. Manage pulseless VT by following advanced cardiac life support (ACLS)
5. Recognize and address TdP
6. Provide care after return of spontaneous circulation (ROSC)
7. Consult intensivist and admit to intensive care unit (ICU)

### Linked objectives and methods

Altered mental status is one of the most common presentations to the emergency department (ED). The patient presents with AMS and is unable to provide any further information; learners are expected to formulate a broad differential diagnosis and an appropriate work-up based on those differentials (Objective #1). An ECG is a part of an AMS work-up. The patient’s ECG showed signs of hypokalemia which the learners are to recognize (Objective #2). Whenever a patient is found to be hypokalemic, hypomagnesemia often accompanies it. Learners should also assess and treat for magnesium abnormalities (Objective #3). As the learners discuss and order electrolyte repletion, the patient goes into TdP on the monitor. Learners should promptly recognize this pattern and initiate ACLS following pulseless VT rhythm (Objective #4). In addition, they will need to treat for TdP (Objective #5). After ROSC, learners will need to provide post-ROSC care (Objective #6). Patient will then need to be admitted to the medical ICU (Objective #7).

### Recommended pre-reading for instructor

1. Farkas J. Torsade de Pointes. EMCrit Project. Published December 1, 2021. Accessed March 4, 2022. At: https://emcrit.org/ibcc/tdp/
2. Simon E, Koyfman A, Long B. EM@3AM - altered mental status. emDOCs.net - Emergency Medicine Education. Published April 7, 2017. Accessed March 4, 2022. At: http://www.emdocs.net/em3am-altered-mental-status/
3. Morgenstern J. Torsades de Pointes: Approach to resuscitation. First10EM. Published February 18, 2019. Accessed March 4, 2022. At: https://first10em.com/torsades-de-pointes/
4. Munro PT, Graham CA. Torsade de pointes. *Emergency Medicine Journal.* 2002; 19**:** 485–486.
5. Farkas J. Hypomagnesemia. EMCrit Project. Published November 28, 2021. Accessed March 4, 2022. At: https://emcrit.org/ibcc/hypomagnesemia/
6. Huang C-L, Kuo E. Mechanism of hypokalemia in magnesium deficiency. *Journal of the American Society of Nephrology*. 2007;18(10):2649–2652. doi:10.1681/asn.2007070792

### Results and tips for successful implementation

This session was conducted on a total of 32 EM interns—a total of 6 interns managed the case while the rest were observers. One actor served as a nurse. Allowing the team to assign roles prior to starting the case helped in running the case smoothly. Depending on the level of the learners, prompting by the nurse may be required to notify them that the patient has an abnormal heart rhythm and no longer has a pulse. When this case was run, the cardiac monitor could be programmed to show torsades. However, if the cardiac monitor is unable to show torsades, a torsades rhythm strip was added to the stimuli to show to the learners. If the learners do not shock the patient during ACLS, prompting to defibrillate and to give magnesium will be required to obtain ROSC. After ROSC, patient will remain altered and should prompt intubation. Novice learners may need guidance by nurse consultant to administer magnesium if not done already.

After the simulation and debriefing session was complete, an online survey was sent via surveymonkey.com to all 32 participants. The responses were collected on a Likert scale of 1 to 5 with 1 being “Strongly disagree” and 5 being “Strongly agree.” The survey collected responses to the following statements:

1. The case was believable.
2. The case had the right amount of complexity.
3. The case helped in improving medical knowledge and patient care.
4. The simulation environment gave me a real-life experience.
5. The debriefing session after simulation helped improve my knowledge.

There was a total of 19 respondents from both years. One hundred percent of them either agreed or strongly agreed that the case was beneficial in learning, improving medical knowledge and patient care. All of them found the post-session debrief to be very helpful. Two of them felt neutral about the case being realistic. The results are shown as a graph below (Chart 1)

**Figure:**
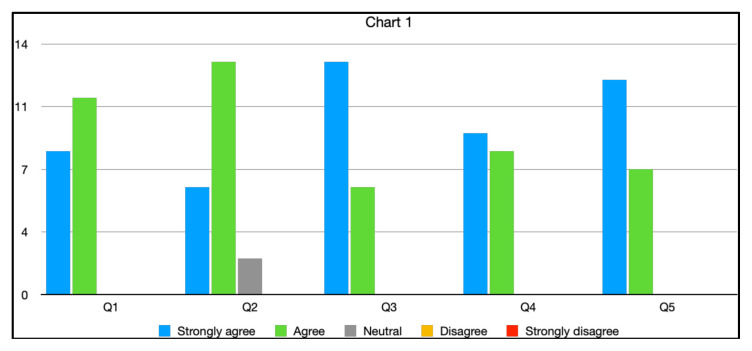


All the comments received from the survey:

“Very believable case that made you synthesize all of the data points that were gathered during the assessment. Great learning experience!”
“Great case”
“It was a great case and learning environment.”
“Excellent case! I appreciated the progression of medical complexity.”
“Great case, really enjoyed it.”

## Supplementary Information



























## Figures and Tables

**Table:** 

#1	Electrocardiogram (ECG) - QTc 520, U waves, Flattened T waves
#2	Complete blood count (CBC)
#3	Basic Metabolic Panel (BMP)
#4	Lactic acid and magnesium
#5	Serum toxicologic drug screen
#6	Urinalysis (UA)
#7	Urine drug screen (UDS)
#8	CT head – normal
#9	Post-intubation chest X-ray (CXR)
#10	ECG strip – torsades de pointes
#11	Post-ROSC ECG – sinus tachycardia

**Table:** 

**Vitals:**	Heart Rate (HR) 105	Blood Pressure (BP) 139/91	Respiratory Rate (RR) 20
	Temperature (T) 36.6°C	Oxygen Saturation (O_2_Sat) 94% on room air	

**Figure:**
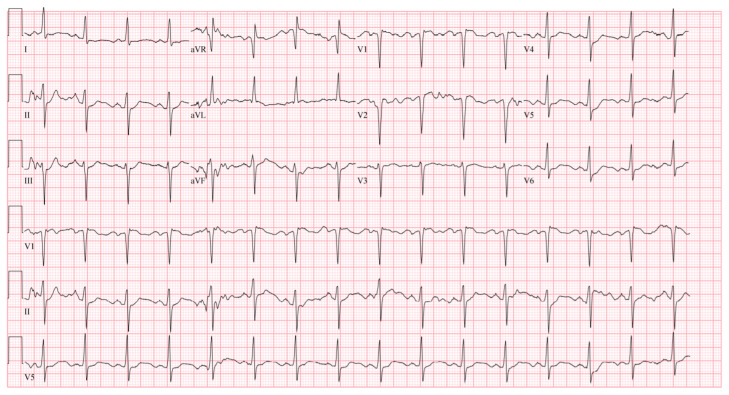


**Table:** 

*Complete blood count (CBC)*	
White blood count (WBC)	13.3 x1000/mm^3^
Hemoglobin (Hgb)	12.7 g/dL
Hematocrit (HCT)	35.7%
Platelet (Plt)	348 x1000/mm^3^

**Table:** 

*Basic metabolic panel (BMP)*	
Sodium	140 mEq/L
Chloride	102 mEq/L
Potassium	2.9 mEq/L
Bicarbonate (HCO_3_)	24 mEq/L
Blood Urea Nitrogen (BUN)	51 mg/dL
Creatine (Cr)	0.71 mg/dL
Glucose	177 mg/dL
Calcium	8.7 mg/dL
*Lactate*	3.1 mg/dL

**Table:** 

*Magnesium*	0.9 mEq/L

**Table:** 

*Serum Toxicologic Drug Screen*	
Ethanol	negative
Salicylate	negative
Acetaminophen	negative

**Table:** 

*Urinalysis (UA)*	
Color	yellow
Appearance	clear
Specific gravity	1.10
pH	7.0
Glucose	negative
Bilirubin	negative
Ketones	2+
Protein	2+
Leukocyte esterase	negative
Nitrites	negative
White blood cells (WBC)	0–5 cells/high powered field (HPF)
Red blood cells (RBC)	0–5 cells/HPF
Squamous epithelial cells	0–5 cells/HPF

**Table:** 

*Urine Toxicology Screen:*	
Amphetamines	negative
Barbiturates	negative
Benzodiazepines	negative
Cocaine	negative
Marijuana	negative
Methadone	negative
Methamphetamine	positive
Opiates	negative
Phencyclidine (PCP)	negative
Tricyclic Antidepressants	negative

**Figure:**
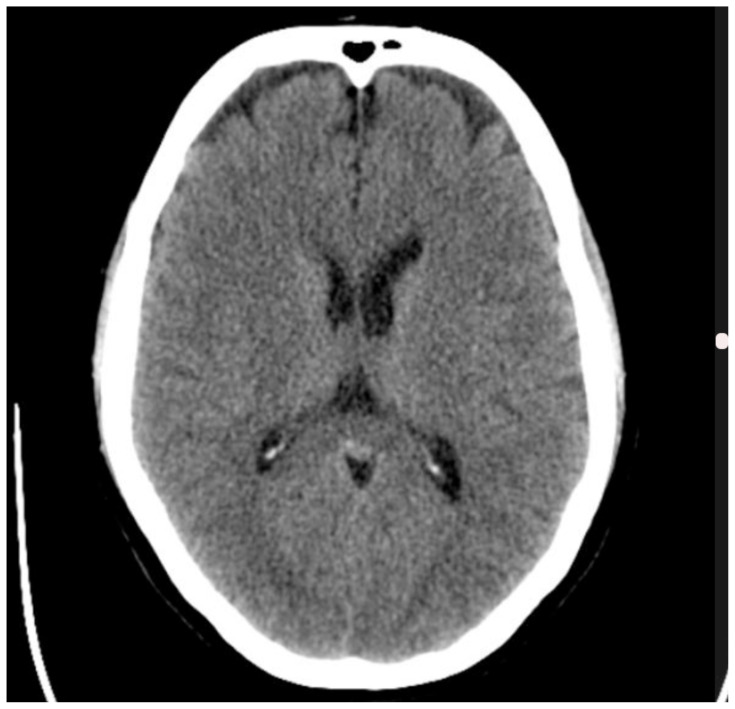


**Figure:**
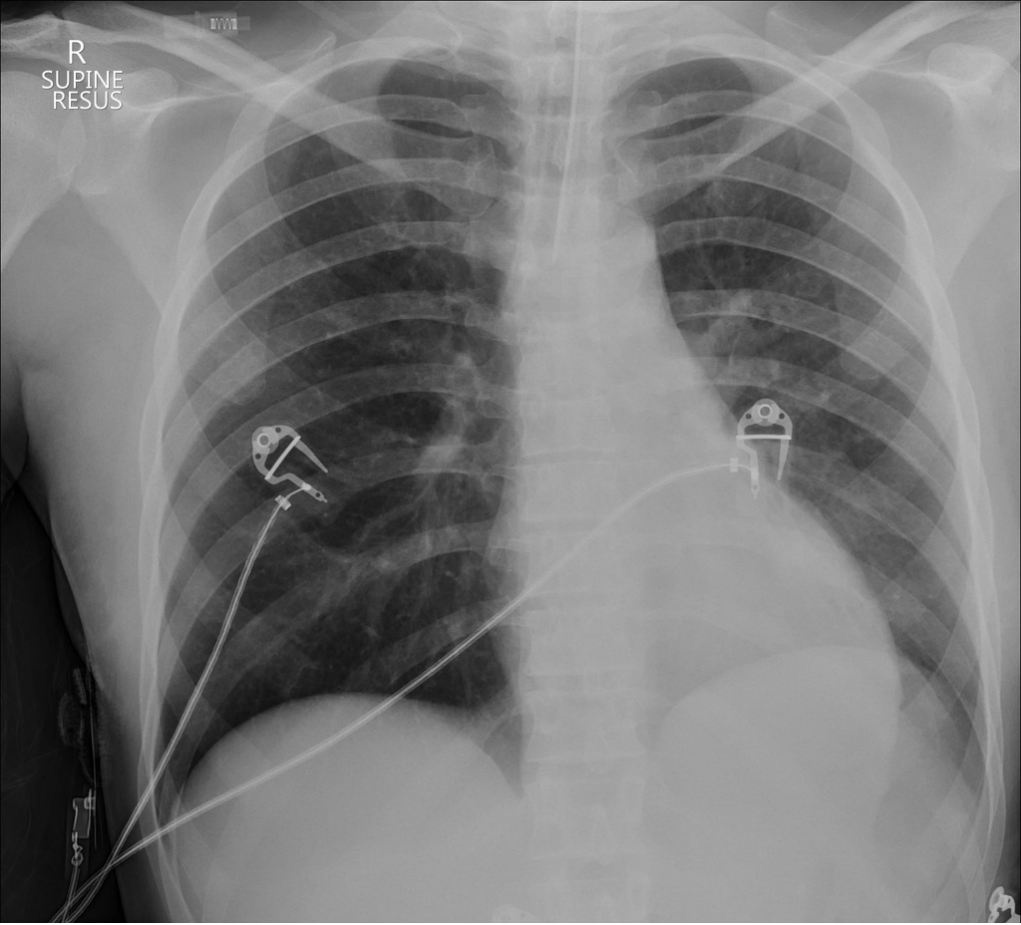


**Figure:**
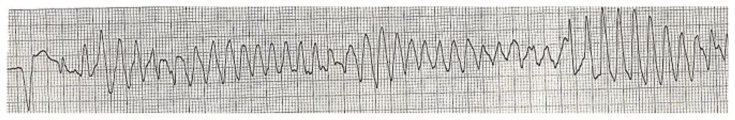


**Figure:**
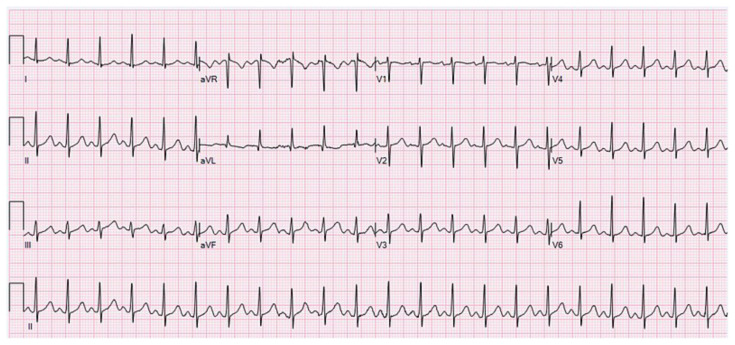


**Table:** SIMULATION EVENTS TABLE:

Minute (state)	Participant action/trigger	Patient status (simulator response) & operator prompts	Monitor display (vital signs)
0:00 (Baseline)	EMS report Team assess vital signs, obtains history and performs a physical exam including assessing neurological status.	Patient mumbling a few words and not able to provide history. Allow them to uncover track marks on own.	T 36.6°C HR 105 BP 139/91 RR 20 O2 94% on RA
2:00–4:00	Team orders ECG, imaging and laboratory studies.	ECG will be provided. Nurse may ask, “Is the ECG normal?”	T 36.6°C HR 110 BP 145/90 RR 18 O2 98% on 2L
4:00–5:00	Team may order IV fluids. Assess stability of the patient prior to going to CT.	Patient continues to be altered and starts to dry heave. Nurse may ask, “Can I take the patient to CT?”	T 36.6°C HR 107 BP 140/85 RR 18 O2 98% on 2L
5:00–7:00	Interpret lab results. Order potassium and magnesium replacement.	Patient continues to be altered but stable. Laboratory results are provided. As the replacement orders are being placed, patient loses pulses.	T 36.6°C HR 95 BP 166/85 RR 20 O2 98% on RA
7:00–10:00	Note the change in the cardiac rhythm. Initiate ACLS. Administer magnesium.	Patient no longer responding and obtunded. Patient loses pulses. Nurse may say, “The patient is not responding anymore and there are no pulses.” Learners will need to be specific in medications and defibrillation orders.	T 36°C HR 200 BP 85/50 RR 24 O2 91% on RA
10:00–12:00	Learners will need to establish endotracheal intubation.	Patient altered and not responding to pain stimulus. After magnesium replacement, patient will get ROSC. Learners will to be establish airway.	T 36°C HR 110 BP 108/63 RR vent O2 99% on ventilator
12:00–14:00	Provide post-ROSC care including ordering ECG.	Patient intubated and on ventilator. Provide ECG and CXR.	T 36°C HR 105 BP 110/70 RR vent O2 99% on ventilator
14:00 – 15:00	Discuss the case with intensivist. Admit to medical ICU.	Intensivist will agree to admit to medical ICU.	T 36°C HR 108 BP 105/65 RR vent O2 99% on ventilator

**Table:** 

**Critical Actions:** Obtain a thorough history and perform a complete physical examination Assess airway, breathing and circulation Broad AMS workup Obtain CT head Laboratory studies including electrolytes, BUN, liver enzymes and ammonia Serum toxicological studies including ethanol, acetaminophen, salicylate levels Urine toxicological studies Analyze ECG and address electrolytes Recognize pulseless VT cardiac arrest Initiate and follow ACLS protocol for VT Treat for TdP by administering magnesium Establish endotracheal intubation Provide post-ROSC care Consult intensivist and admit to ICU	0:00

**Table:** 

	Milestone	Did not achieve level 1	Level 1	Level 2	Level 3
1	**Emergency Stabilization (PC1)**	□ Did not achieve Level 1	□ Recognizes abnormal vital signs	□ Recognizes an unstable patient, requiring intervention Performs primary assessment Discerns data to formulate a diagnostic impression/plan	□ Manages and prioritizes critical actions in a critically ill patient Reassesses after implementing a stabilizing intervention
2	**Performance of focused history and physical (PC2)**	□ Did not achieve Level 1	□ Performs a reliable, comprehensive history and physical exam	□ Performs and communicates a focused history and physical exam based on chief complaint and urgent issues	□ Prioritizes essential components of history and physical exam given dynamic circumstances
3	**Diagnostic studies (PC3)**	□ Did not achieve Level 1	□ Determines the necessity of diagnostic studies	□ Orders appropriate diagnostic studies. Performs appropriate bedside diagnostic studies/procedures	□ Prioritizes essential testing Interprets results of diagnostic studies Reviews risks, benefits, contraindications, and alternatives to a diagnostic study or procedure
4	**Diagnosis (PC4)**	□ Did not achieve Level 1	□ Considers a list of potential diagnoses	□ Considers an appropriate list of potential diagnosis May or may not make correct diagnosis	□ Makes the appropriate diagnosis Considers other potential diagnoses, avoiding premature closure
5	**Pharmacotherapy (PC5)**	□ Did not achieve Level 1	□ Asks patient for drug allergies	□ Selects an medication for therapeutic intervention, consider potential adverse effects	□ Selects the most appropriate medication and understands mechanism of action, effect, and potential side effects Considers and recognizes drug-drug interactions
6	**Observation and reassessment (PC6)**	□ Did not achieve Level 1	□ Reevaluates patient at least one time during case	□ Reevaluates patient after most therapeutic interventions	□ onsistently evaluates the effectiveness of therapies at appropriate intervals
7	**Disposition (PC7)**	□ Did not achieve Level 1	□ Appropriately selects whether to admit or discharge the patient	□ Appropriately selects whether to admit or discharge Involves the expertise of some of the appropriate specialists	□ Educates the patient appropriately about their disposition Assigns patient to an appropriate level of care (ICU/Tele/Floor) Involves expertise of all appropriate specialists
9	**General Approach to Procedures (PC9)**	□ Did not achieve Level 1	□ Identifies pertinent anatomy and physiology for a procedure Uses appropriate Universal Precautions	□ Obtains informed consent Knows indications, contraindications, anatomic landmarks, equipment, anesthetic and procedural technique, and potential complications for common ED procedures	□ Determines a back-up strategy if initial attempts are unsuccessful Correctly interprets results of diagnostic procedure
20	**Professional Values (PROF1)**	□ Did not achieve Level 1	□ Demonstrates caring, honest behavior	□ Exhibits compassion, respect, sensitivity and responsiveness	□ Develops alternative care plans when patients’ personal beliefs and decisions preclude standard care
22	**Patient centered communication (ICS1)**	□ Did not achieve level 1	□ Establishes rapport and demonstrates empathy to patient (and family) Listens effectively	□ Elicits patient’s reason for seeking health care	□ Manages patient expectations in a manner that minimizes potential for stress, conflict, and misunderstanding. Effectively communicates with vulnerable populations, (at risk patients and families)
23	**Team management (ICS2)**	□ Did not achieve level 1	□ Recognizes other members of the patient care team during case (nurse, techs)	□ Communicates pertinent information to other healthcare colleagues	□ Communicates a clear, succinct, and appropriate handoff with specialists and other colleagues Communicates effectively with ancillary staff

## INSTRUCTOR MATERIALS

**Case Title:** Torsade de Pointes Due to Hypokalemia and Hypomagnesemia

**Case Description & Diagnosis (short synopsis):** A 45-year-old female with past medical history of IV methamphetamine (meth) use brought in by emergency medical services (EMS) for altered mental status.

Per EMS report, patient’s boyfriend called EMS because he was concerned about patient not responding properly. Patient was found be sitting upright and alert and oriented x4. She did have slurred speech and was slow to respond.

Per chart review, patient does have history of IV meth use and similar presentations to this emergency department.

In the emergency department (ED), patient is alert and oriented x 1 and follows some commands. ECG shows signs of hypokalemia, in which a magnesium level should added. As laboratory results are given, the patient loses pulses and will be in TdP. ACLS should be initiated, and the patient should be promptly defibrillated. Magnesium should be administered and ROSC will be obtained. Despite ROSC, patient remains altered prompting endotracheal intubation and intensive care unit (ICU) admission

**Equipment or Props Needed:**

– High-fidelity simulator
– Track marks on bilateral forearms
– Airway supplies:
  ○ Nasal cannula
  ○ Non-rebreather mask
  ○ Laryngoscope and blades
  ○ Endotracheal tube and stylet
  ○ Bag-valve mask
– IV supplies:
  ○ 2 18g angiocatheters
  ○ IV tubing
  ○ Normal saline
  ○ Lactated ringers
– Medications:
  ○ Etomidate
  ○ Succinylcholine
  ○ Epinephrine
  ○ Sodium bicarbonate
  ○ Magnesium sulfate
  ○ Potassium Chloride
  ○ Amiodarone
  ○ Lidocaine
– Cardiac monitor
– Defibrillator
– Gloves

**Confederates needed:**

Operator may play nurse or EMS

**Stimulus Inventory:**

**Background and brief information:** A 45-year-old female with past medical history of IV meth use brought in by EMS for altered mental status.

**Initial presentation:** EMS report: patient’s boyfriend called EMS because he was concerned about patient not responding properly. Patient was found be sitting upright and alert and oriented x4. She did have slurred speech and was slow to respond.

Per chart review, patient does have history of IV meth use and similar presentations to this emergency department.

In the emergency department (ED), patient is alert and oriented x 1 and follows some commands.

**How the scene unfolds:** Upon arrival to the ED, the learners should start by assessing the patient’s mental status. They may ask for what history is found after chart review, and they will be provided with the history of patient using IV meth and multiple presentations to the ED. Learners may order laboratory studies to include the broad differential for altered mental status and order ECG and CT head. ECG will be provided as the patient goes to the CT head. Learners may recognize the U waves, T wave flattening, and long QTc. After patient returns from CT, learners will be given the lab results and the CT head. Learners may recognize the hypokalemia and hypomagnesemia and order repletion. As the repletion orders are placed, patient will lose pulses and go into ventricular tachycardia with rhythm looking like torsades de pointes. Learners will need to initiate ACLS and defibrillate. They will also need to replace magnesium quickly. After magnesium replacement, patient will get ROSC. Despite ROSC, patient will continue to not respond to pain stimuli prompting intubation. After endotracheal intubation, learners will provide post-ROSC care including ordering post-intubation chest X-ray and ECG. After interpreting them, they will need to discuss the case with intensivist for admission to medical ICU.

**Critical actions:**

1. Obtain a thorough history and perform a complete physical examination
2. Assess airway, breathing, and circulation
3. Broad AMS workup
  - Obtain CT head
  - Laboratory studies including electrolytes, BUN, liver enzymes and ammonia
  - Serum toxicological studies including ethanol, acetaminophen salicylate levels
  - Urine toxicological studies
4. Analyze ECG and address electrolytes
5. Recognize pulseless VT cardiac arrest
6. Initiate and follow ACLS protocol for VT
7. Treat for TdP by administering magnesium
8. Establish endotracheal intubation
9. Provide post-ROSC care
10. Consult intensivist and admit to ICU

**Case Title:** Torsade de Pointes Due to Hypokalemia and Hypomagnesemia

**Chief Complaint:** “Not responding properly”

**General Appearance:** alert and oriented x1, follows some commands, mumbling

**Primary Survey:**

- **Airway:** mumbling a few words
- **Breathing:** Clear to auscultation bilaterally. No rales or rhonchi
- **Circulation:** Tachycardic with bilateral radial and dorsalis pedis pulses present

**History:**

- **History of present illness:** EMS report: patient’s boyfriend called EMS because he was concerned about patient not responding properly. Patient was found be sitting upright and alert and oriented x 1. She did have slurred speech and was slow to respond. They placed an IV and started fluids on her.
- Per chart review, patient does have history of IV meth use and similar presentations to this emergency department.
- Further history including review of systems cannot be obtained at this time due to patient’s mental status

**Past medical history:** Per chart review, none

**Past surgical history:** Per chart review, none

**Patient’s medications:** Unknown

**Allergies:** none

**Social history:** Per chart review, methamphetamine abuse

**Family history:** Unknown

**Secondary Survey/Physical Examination:**

- **General appearance:** alert and oriented x1, follows some commands, mumbling
- **HEENT:**
  ○ **Head:** Within normal limits
  ○ **Eyes:** Pupils are 4mm, round and reflective to light
  ○ **Ears:** Within normal limits
  ○ **Nose:** Within normal limits
  ○ **Throat:** dry mucous membranes
- **Neck:** No thyromegaly
- **Heart:** S1 and S2 normal but tachycardic. No murmurs or gallops
- **Lungs:** clear to auscultation bilaterally. No rales or rhonchi
- **Abdominal/GI:** Soft, non-tender, nondistended. No scars present. No rigidity, guarding, or rebound tenderness
- **Genitourinary:** Within normal limits
- **Rectal:** deferred
- **Extremities:** Multiple bruises that appear like track marks noted on the anterior aspects of her arms bilaterally
- **Back:** Within normal limits
- **Neuro:** follows commands, no focal neurologic deficits, no cranial nerve deficits
- **Skin:** Multiple bruises that appear like track marks noted on the anterior aspects of her arms bilaterally
- **Lymph:** Within normal limits
- **Psych:** Within normal limits

**Results:**

*Electrocardiogram (ECG) –* QTc 520, U waves, Flattened T waves

Image source: (author’s own image)

*CT head –* Normal

Image source: Author’s own image

*Post-intubation CXR –* ET tube in good position

Smith D. Endotracheal tube in right main bronchus. In: Radiopaedia. https://radiopaedia.org/?lang=us>Radiopaedia.org. CC BY-NC-SA 3.0.

*ECG strip –* Torsades

Image source: Torsades converted by AICD ECG strip Lead II. In: Wikimedia Commons. https://commons.wikimedia.org/wiki/File:Torsades_converted_by_AICD_ECG_strip_Lead_II.JPG Public Domain.

*Post-ROSC ECG –* Sinus tachycardia

Image source: Ewingdo. ECG Sinus Tachycardia 132 bpm. In: Wikimedia Commons. https://commons.wikimedia.org/wiki/File:ECG_Sinus_Tachycardia_132_bpm.jpg. CC BY-SA 4.0.

## OPERATOR MATERIALS

**Diagnosis:**

Hypokalemia, Hypomagnesemia, Torsades de Pointes

**Disposition:**

ICU

## DEBRIEFING AND EVALUATION PEARLS

### Hypokalemia, Hypomagnesemia, Torsades de Pointes

#### AMS

The approach to altered mental status (AMS) in the ED should begin with airway, breathing, circulation assessment along with vital signs including a point of care glucose level. To memorize the causes of AMS, one can use the mnemonic AEIOU TIPS: Alcohol, Encephalopathy (hypertensive, hepatic), Electrolytes, Endocrine, Environmental, Insulin (hypoglycemia, HHS, DKA), Opiates, Oxygen (hypoxia), Uremia, Trauma, Toxins, Infection, Increased intracranial pressure, Psychosis, Poisoning (cyanide, carbon monoxide, etc.), Stroke, and Seizure. Work-up for AMS should include EKG, CBC, CMP, UA, toxicologic studies (UDS, salicylates, acetaminophen, ethanol), creatine kinase, and CT head without contrast. Additional labs and imaging dictated by history and physical may include troponin, thyroid, sepsis labs (blood cultures, lactate, urine culture), ammonia, medication levels, CSF evaluation, and CXR.^3^

#### Hypomagnesemia and Hypokalemia

Hypomagnesemia is defined as a serum magnesium level <1.4 mEq/L (<1 mEq/L = severe). Clinical presentations can include neuromuscular signs and symptoms such as irritability/lethargy, tetany, tremor, and hyperreflexia. Low magnesium levels can also lead to cardiovascular effects causing ECG changes such as prolonged PR and QT intervals, wide QRS complex, ST depressions, and broad flat T waves with precordial T wave inversion. Metabolic abnormalities including hypokalemia and hypocalcemia often co-exist. Etiology can stem from inadequate intake or absorption, endocrine disorders, alcoholism, pancreatitis, or drugs (aminoglycosides, amphotericin B, diuretics, β-agonists, and cyclosporine).^4^ In this case, the patient has had inadequate intake due to the chronic methamphetamine use. Magnesium distribution in the body is depicted in the chart, in which a majority is found in the bone, muscle, and soft tissue. Only <5% is found in the serum. Therefore, serum magnesium levels may not accurately reflect the actual magnesium level in the body.^5^ A patient with severe symptoms who is hemodynamically unstable should receive magnesium replacement therapy with MgSO4 as a 2 g bolus over 10 min. For patients with severe symptoms that are hemodynamically stable, administer MgSO4 2 g bolus over 1 hour. For concurrent repletion with potassium, administer a 5 g per hour infusion.^4^

Hypomagnesemia is often accompanied by hypokalemia due to the relation of magnesium and potassium excretion in the renal tubules. Potassium is excreted through the renal outer medullary potassium (ROMK) channels in the renal tubules. Magnesium inhibits the ROMK channels leading to increased potassium levels in the blood. However, if magnesium levels are low, there will be disinhibition of ROMK channels leading to increased potassium excretion and therefore hypokalemia. 5

Hypokalemia is defined as serum potassium level <3.5 mEq/L (<2.5 mEq/L = severe). Hypokalemia may be a result of GI conditions such as vomiting, gastric suctioning, starvation, chronic diarrhea, colon cancer, or licorice ingestion, or renal conditions with metabolic acidosis or metabolic alkalosis, intracellular shifts of glucose/insulin, transcellular shifts due to β-agonists, excess mineralocorticoids (↑ renal excretion of K^+^), sweating, hyperthyroidism, and hypomagnesemia. Clinical presentation can include pronounced weakness, hyporeflexia, paralysis, bradycardia/sinus arrest, first degree AV block, idioventricular rhythm, ventricular tachycardia, ventricular fibrillation, and ileus. Cardiac effects of hypokalemia can lead to a progression of changes on an EKG. T-waves will shrink and invert, an enlarged u-wave will appear, and then there will be QTc prolongation. Potassium repletion of 10 mEq potassium chloride (KCl) will increase the serum potassium level by 0.1 mEq/L. For subacute or chronic hypokalemia, oral KCl should be administered. For acute hypokalemia in which a patient is symptomatic and unable to take oral potassium, administer 10 mEq/hr KCl IV for routine potassium repletion. For severe hypokalemia or DKA, administer 20 mEq/hr IV; however, do not run through a single peripheral IV line to *prevent vein sclerosis*. Rather, run repletion through a central line or split into two different peripheral lines.^6^

#### Torsade de Pointes

Torsades de pointes (“twisting of the points”) is a polymorphic ventricular tachycardia in which the QRS axis swings from a positive to a negative direction in a single lead creating a “sinewave” appearance on ECG associated with a prolonged QT interval. Causes of TdP are things that prolong QT such as hypomagnesemia, hypokalemia, and drugs (Class IA and IC antidysrhythmics, Tricyclic antidepressants, Droperidol, Phenothiazines, Methadone, etc.).^7^

TdP is a ventricular tachycardia, and therefore should be treated as such. Defibrillation is indicated in a patient without a pulse. Synchronized Cardioversion is indicated for an unstable patient with a pulse but is reasonable for a stable patient as well. Medical management of TdP should include a magnesium loading dose of 2 grams IV bolus over 10 min, then start an infusion at 1–4 grams/hr. Ensure to monitor clinically because magnesium can cause hypotension and respiratory depression. Do NOT give amiodarone or procainamide because this may further prolong the QTc and arrhythmia. 7

Once the patient has been converted out of TdP, it is important to treat the underlying cause of the arrhythmia to prevent recurrence. First line treatment is magnesium as described above to stabilize the cardiac membrane. Heart rate and the QT interval have an inverse relationship; therefore, increasing the heart rate will decrease the QT interval and reduce the overall risk of TdP. The goal heart rate is 90–110 beats per minute (bpm) but can go up to as high as 140 bpm. Medical chronotropy is the fastest and easiest way to stabilize the patient. Lidocaine is also a preferred agent in treating TdP; however, there is limited evidence to support its use.^7^ The agent used will depend on the hemodynamic stability of the patient. If the patient is severely hypotensive, start an epinephrine infusion. If they are slightly hypotensive or normotensive, start Isoproterenol at 5 mcg/min and titrate to 30 bpm higher than baseline heart rate or whatever heart rate required to prevent TdP. If medical therapy fails, electrical chronotropy can be achieved with either transcutaneous or transvenous pacing.^8^

## SIMULATION ASSESSMENT

*Torsades de Pointes Due to Hypokalemia and Hypomagnesemia*

Learner: _________________________________________

***Assessment Timeline***

This timeline is to help observers assess their learners. It allows observer to make notes on when learners performed various tasks, which can help guide debriefing discussion.

**Critical Actions:**

□ Obtain a thorough history and perform a complete physical examination
□ Assess airway, breathing and circulation
□ Broad AMS workup
  □ Obtain CT head
  □ Laboratory studies including electrolytes, BUN, liver enzymes and ammonia
  □ Serum toxicological studies including ethanol, acetaminophen, salicylate levels
  □ Urine toxicological studies
□ Analyze ECG and address electrolytes
□ Recognize pulseless VT cardiac arrest
□ Initiate and follow ACLS protocol for VT
□ Treat for TdP by administering magnesium
□ Establish endotracheal intubation
□ Provide post-ROSC care
□ Consult intensivist and admit to ICU

**Summative and formative comments:**

**Milestones assessment:**

## References

[1] Cohagan B Torsade de Pointes. StatPearls [Internet].

[2] McCauley M, Vallabhajosyula S, Darbar D Proarrhythmic and torsadogenic effects of potassium channel blockers in patients. Cardiac electrophysiology clinics.

[3] Simon E, Koyfman A, Long B EM@3AM - altered mental status. emDOCs.net - Emergency Medicine Education.

[4] Farkas J Hypomagnesemia. EMCrit Project.

[5] Huang C-L, Kuo E (2007). Mechanism of hypokalemia in magnesium deficiency. Journal of the American Society of Nephrology.

[6] Farkas J Hypokalemia. EMCrit Project.

[7] Farkas J Torsade de Pointes. EMCrit Project.

[8] Munro PT, Graham CA (2002). Torsade de pointes. Emergency Medicine Journal.

