# Supplementary material for: Torsade de Pointes Due to Hypokalemia and Hypomagnesemia
Source: J Educ Teach Emerg Med. 2022 Oct 15;7(4):S27–51. doi: 10.21980/J8JP8G (PMC10332665; doi:10.21980/J8JP8G)
Supplement: Supplementary file 1 [file JETem-7-4-S27-supp1.pptx]

## Slide 1
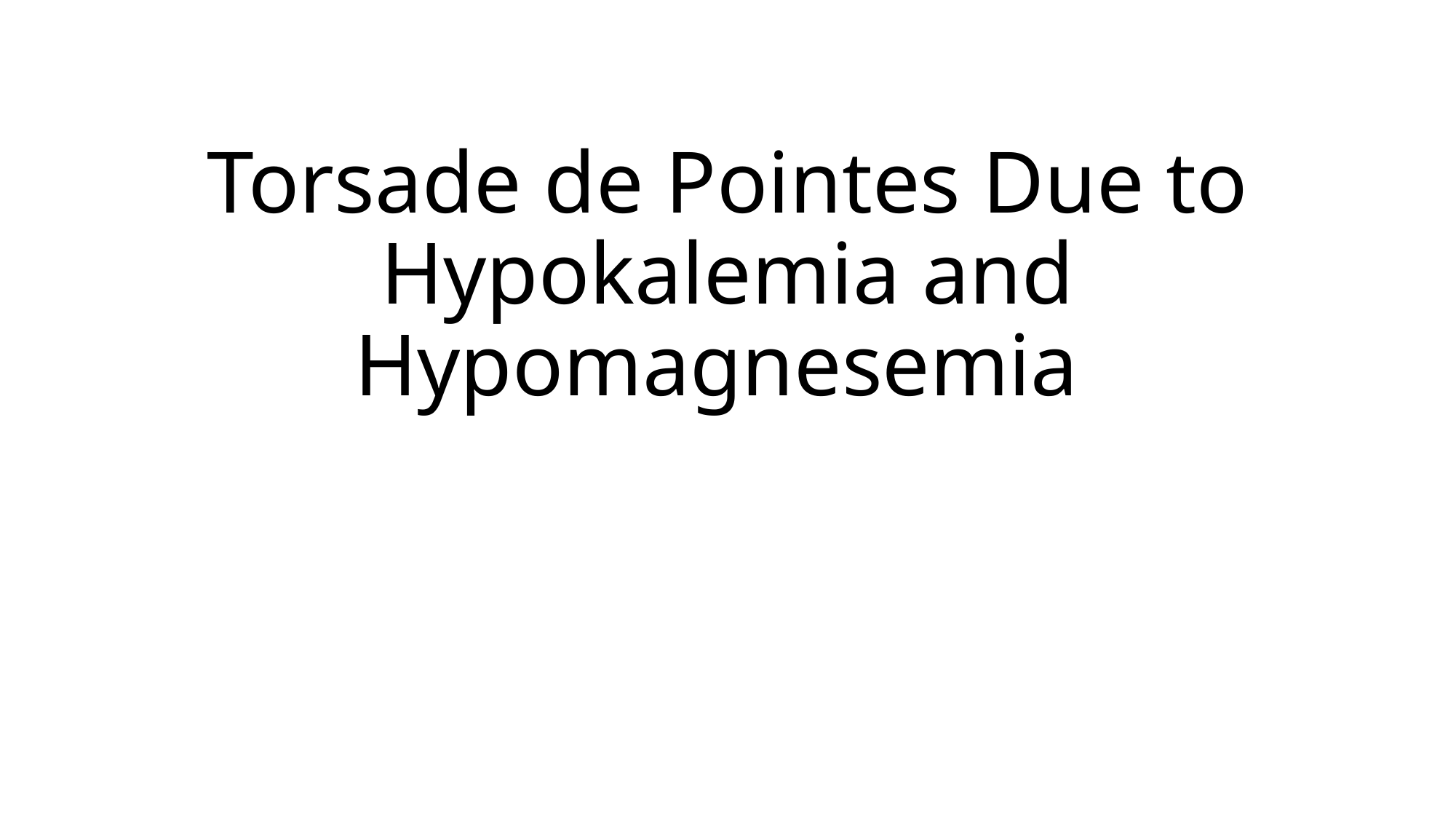

# Torsade de Pointes Due to Hypokalemia and Hypomagnesemia

## Slide 2
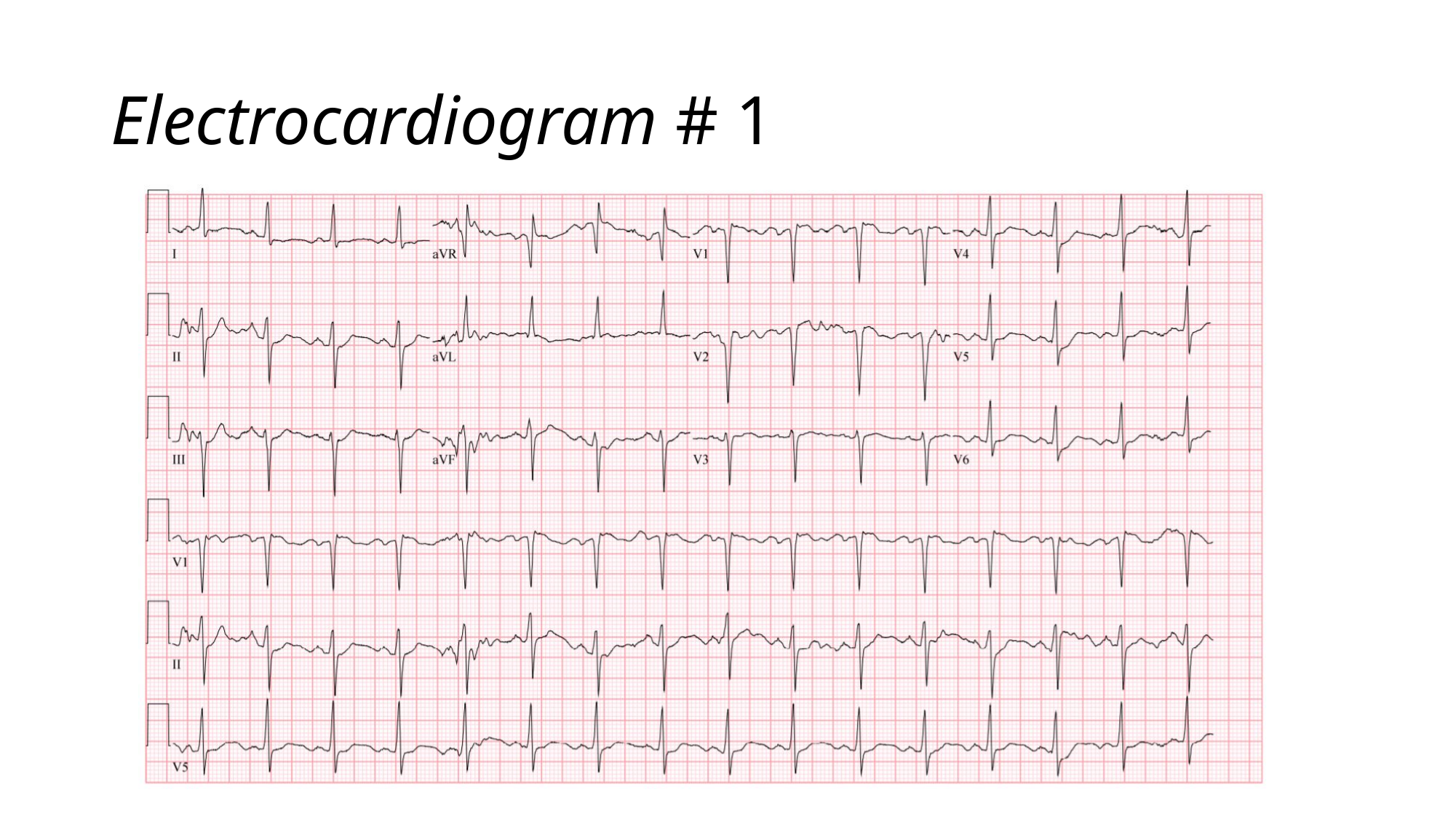

# Electrocardiogram # 1

## Slide 3
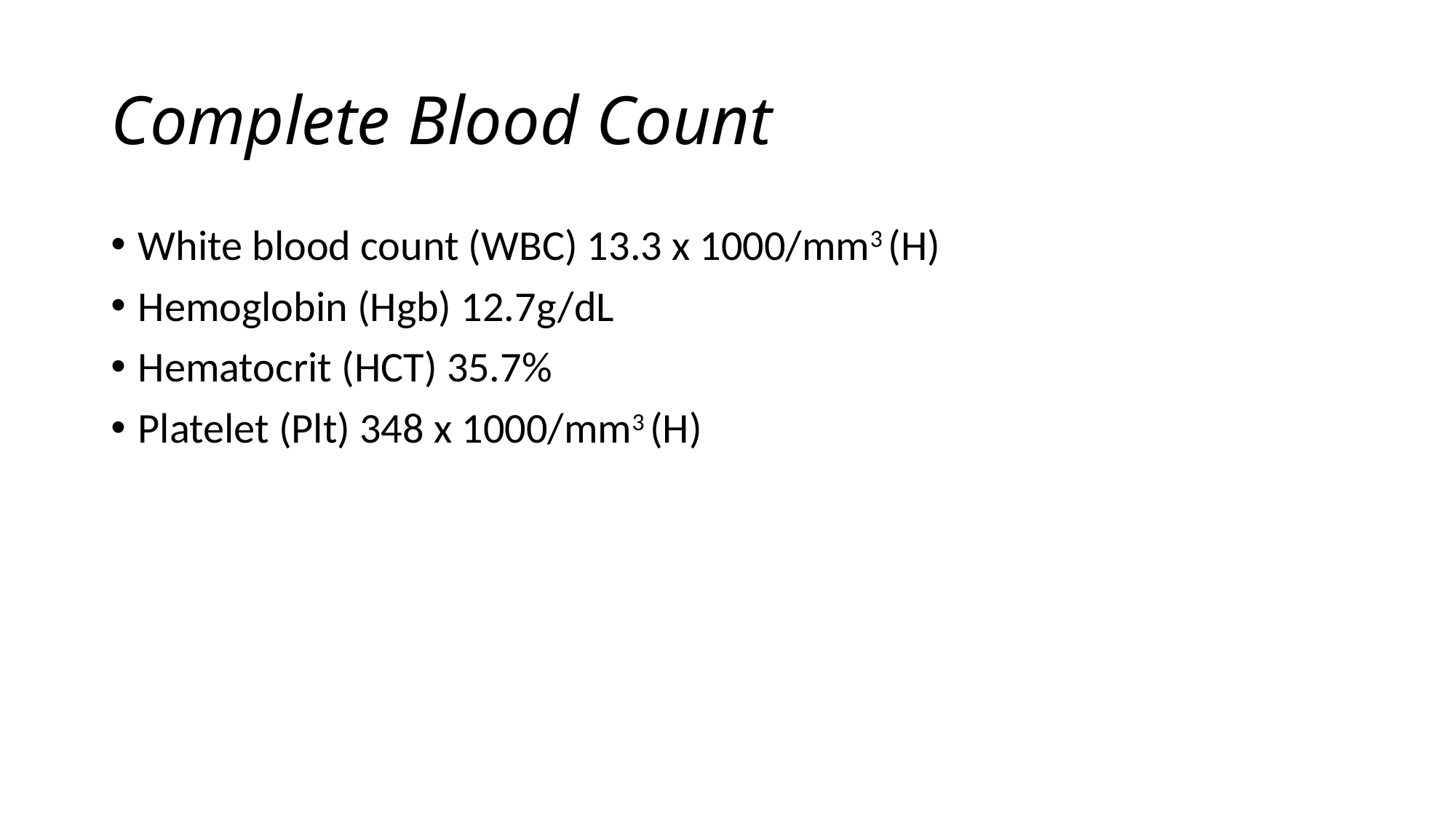

# Complete Blood Count
White blood count (WBC) 13.3 x 1000/mm3 (H)
Hemoglobin (Hgb) 12.7g/dL
Hematocrit (HCT) 35.7%
Platelet (Plt) 348 x 1000/mm3 (H)

## Slide 4
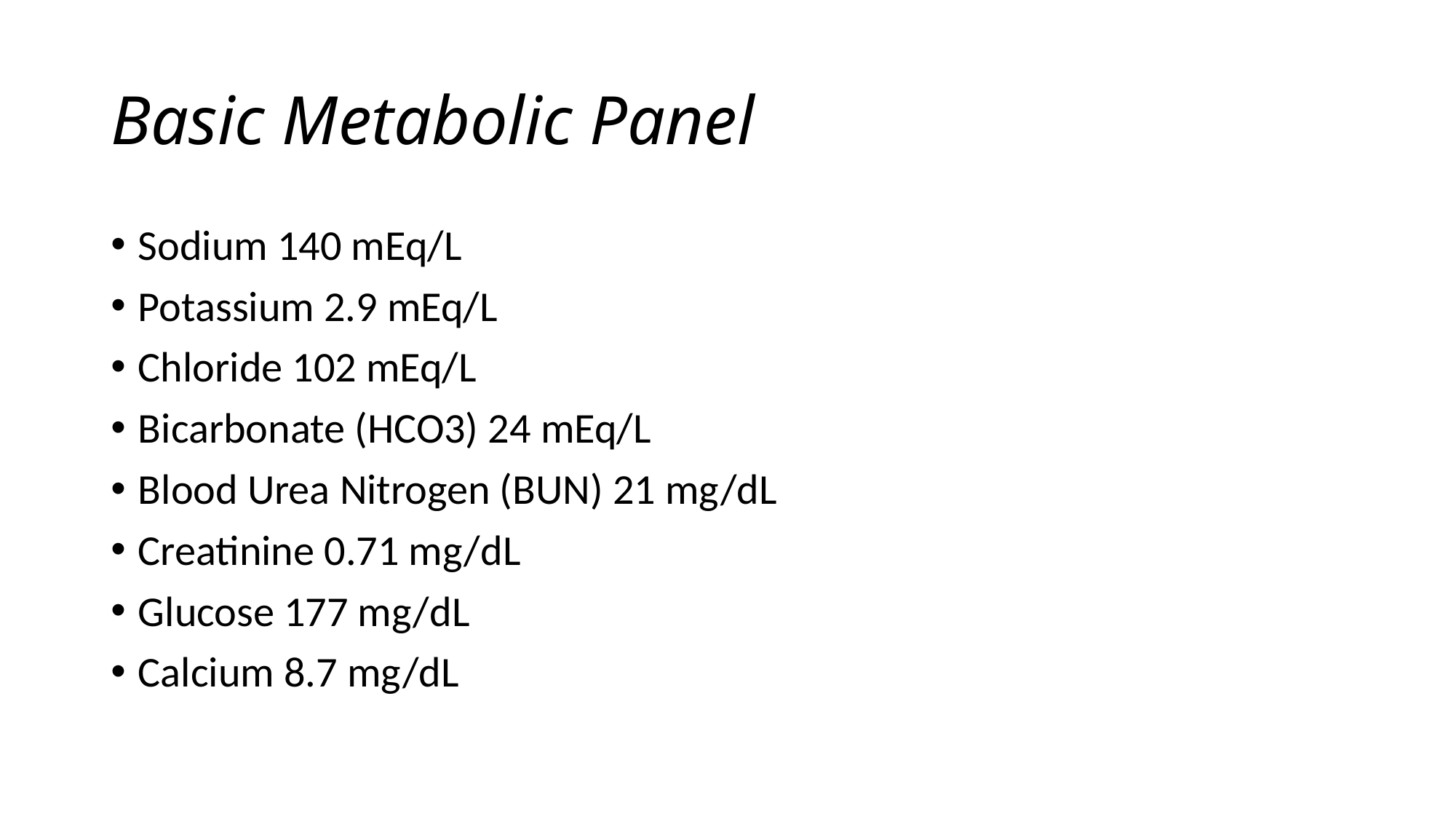

# Basic Metabolic Panel
Sodium 140 mEq/L
Potassium 2.9 mEq/L
Chloride 102 mEq/L
Bicarbonate (HCO3) 24 mEq/L
Blood Urea Nitrogen (BUN) 21 mg/dL
Creatinine 0.71 mg/dL
Glucose 177 mg/dL
Calcium 8.7 mg/dL

## Slide 5
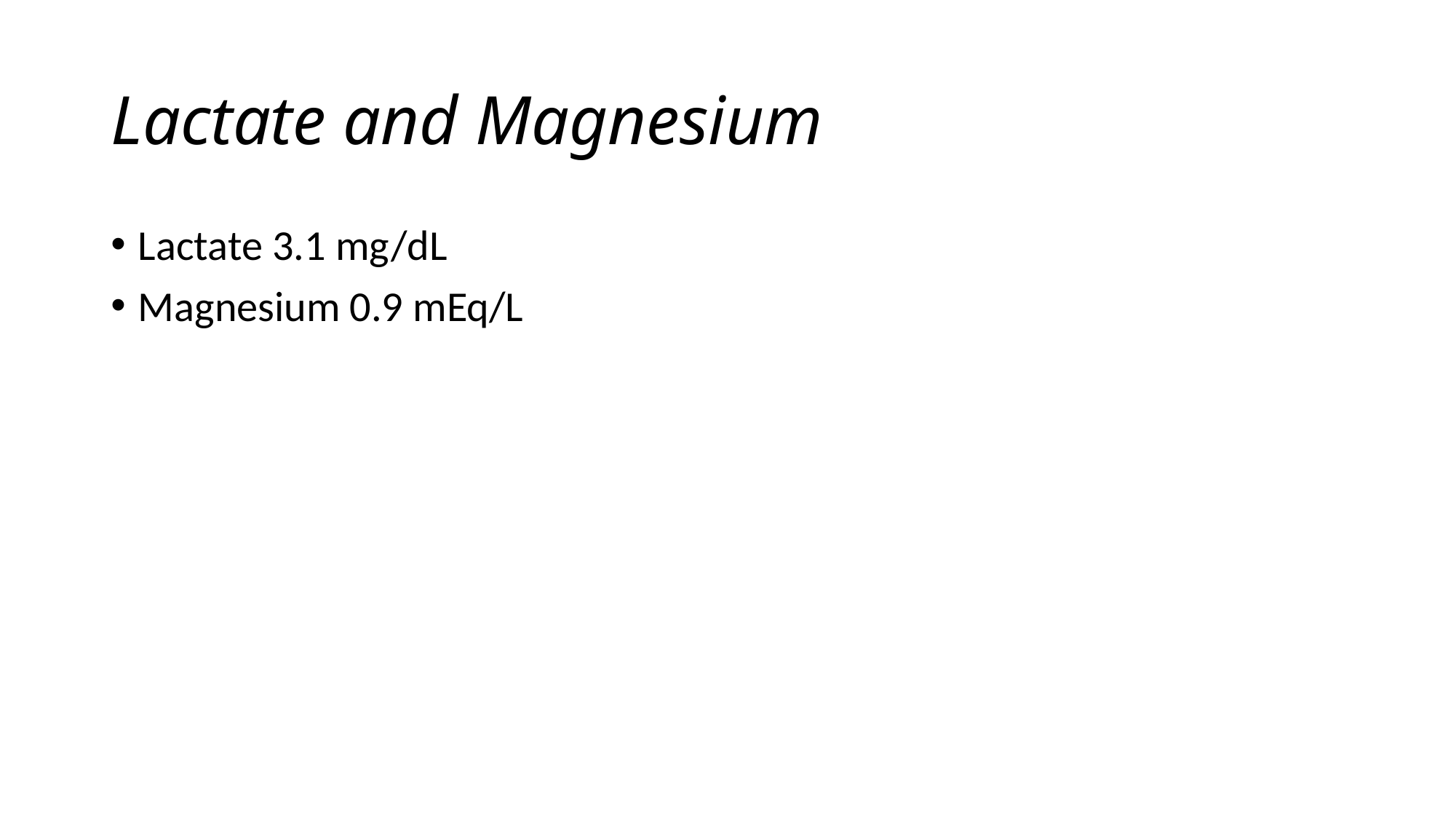

# Lactate and Magnesium
Lactate 3.1 mg/dL
Magnesium 0.9 mEq/L

## Slide 6
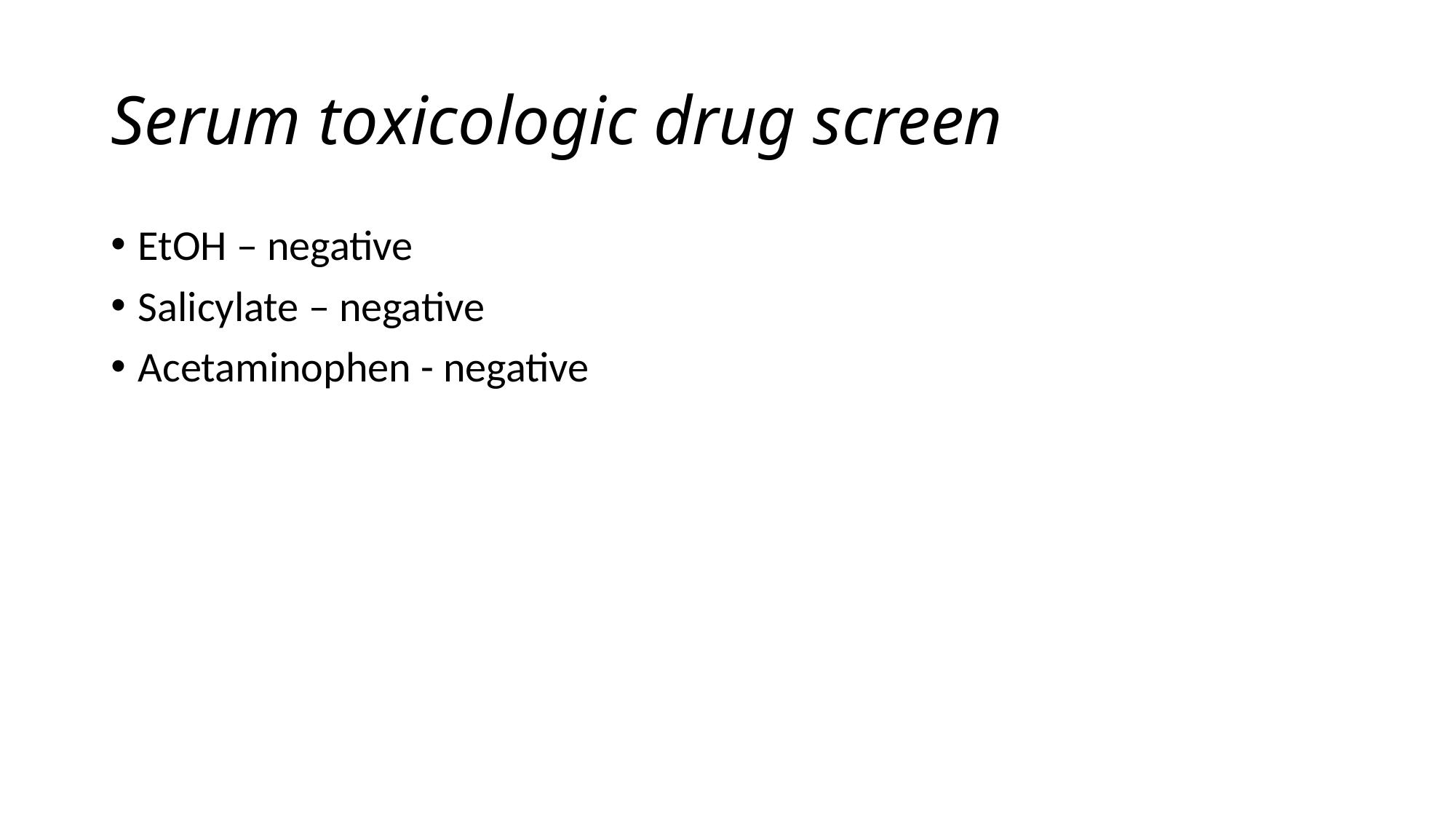

# Serum toxicologic drug screen
EtOH – negative
Salicylate – negative
Acetaminophen - negative

## Slide 7
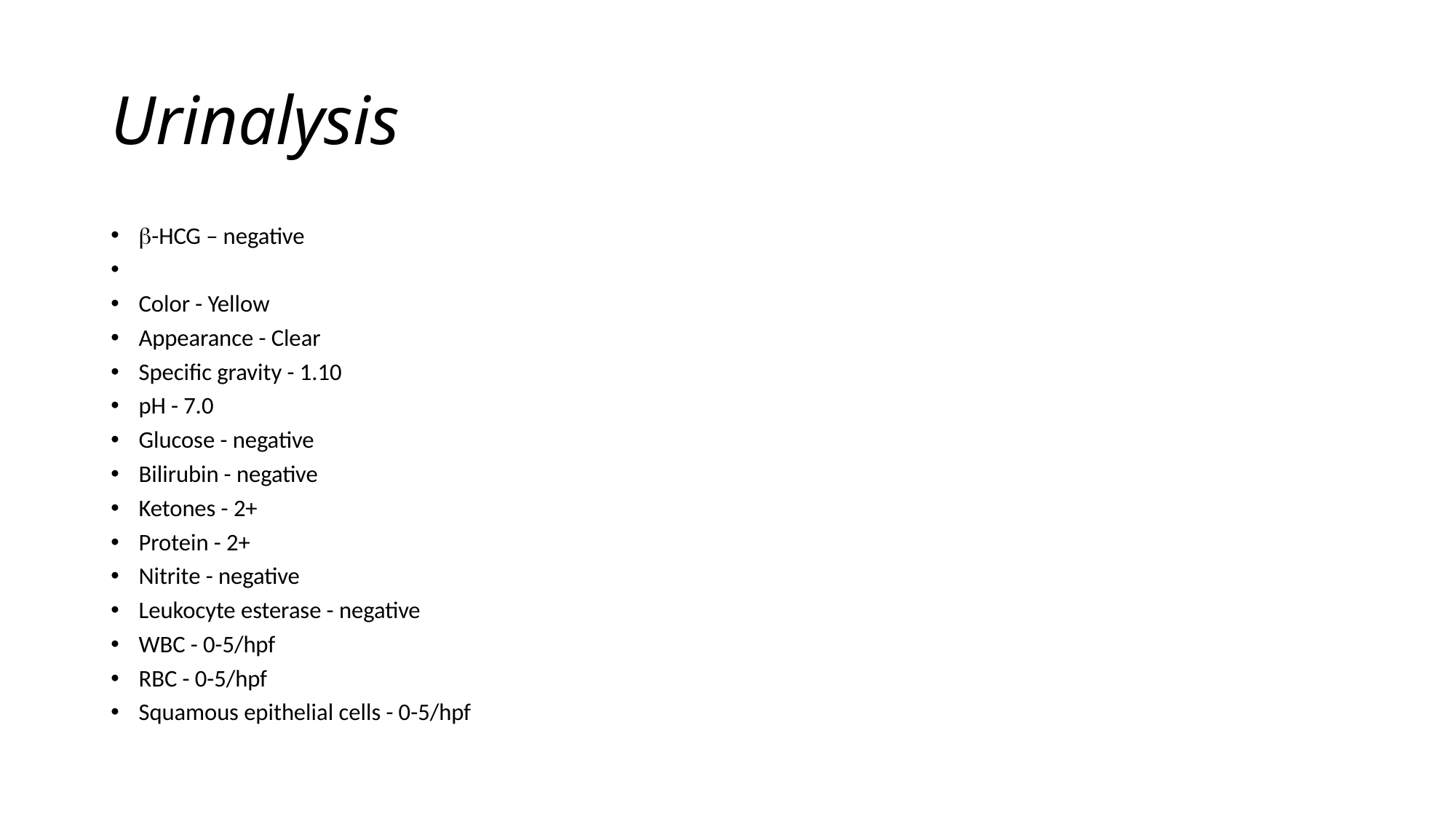

# Urinalysis
β-HCG – negative
Color - Yellow
Appearance - Clear
Specific gravity - 1.10
pH - 7.0
Glucose - negative
Bilirubin - negative
Ketones - 2+
Protein - 2+
Nitrite - negative
Leukocyte esterase - negative
WBC - 0-5/hpf
RBC - 0-5/hpf
Squamous epithelial cells - 0-5/hpf

## Slide 8
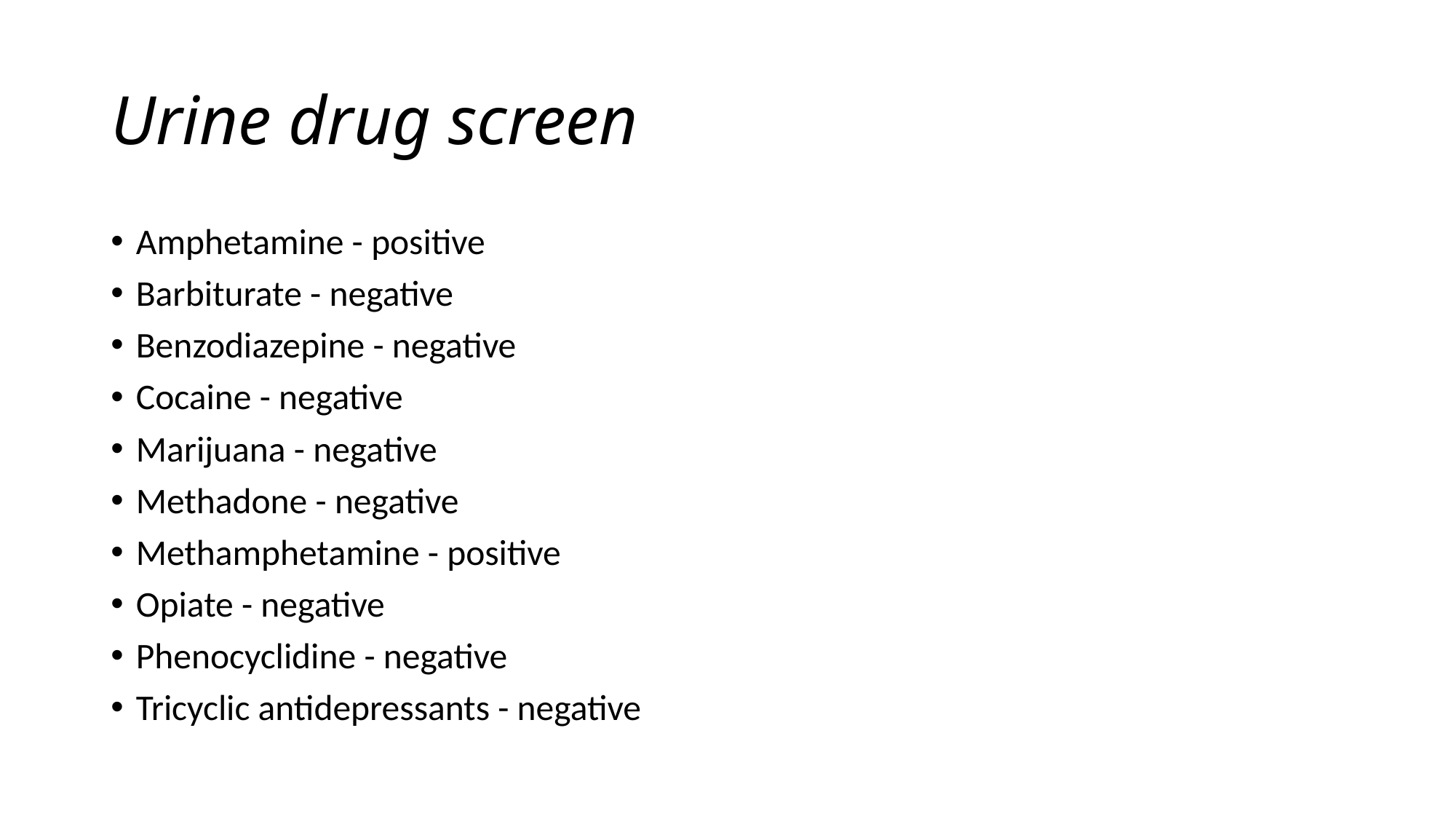

# Urine drug screen
Amphetamine - positive
Barbiturate - negative
Benzodiazepine - negative
Cocaine - negative
Marijuana - negative
Methadone - negative
Methamphetamine - positive
Opiate - negative
Phenocyclidine - negative
Tricyclic antidepressants - negative

## Slide 9
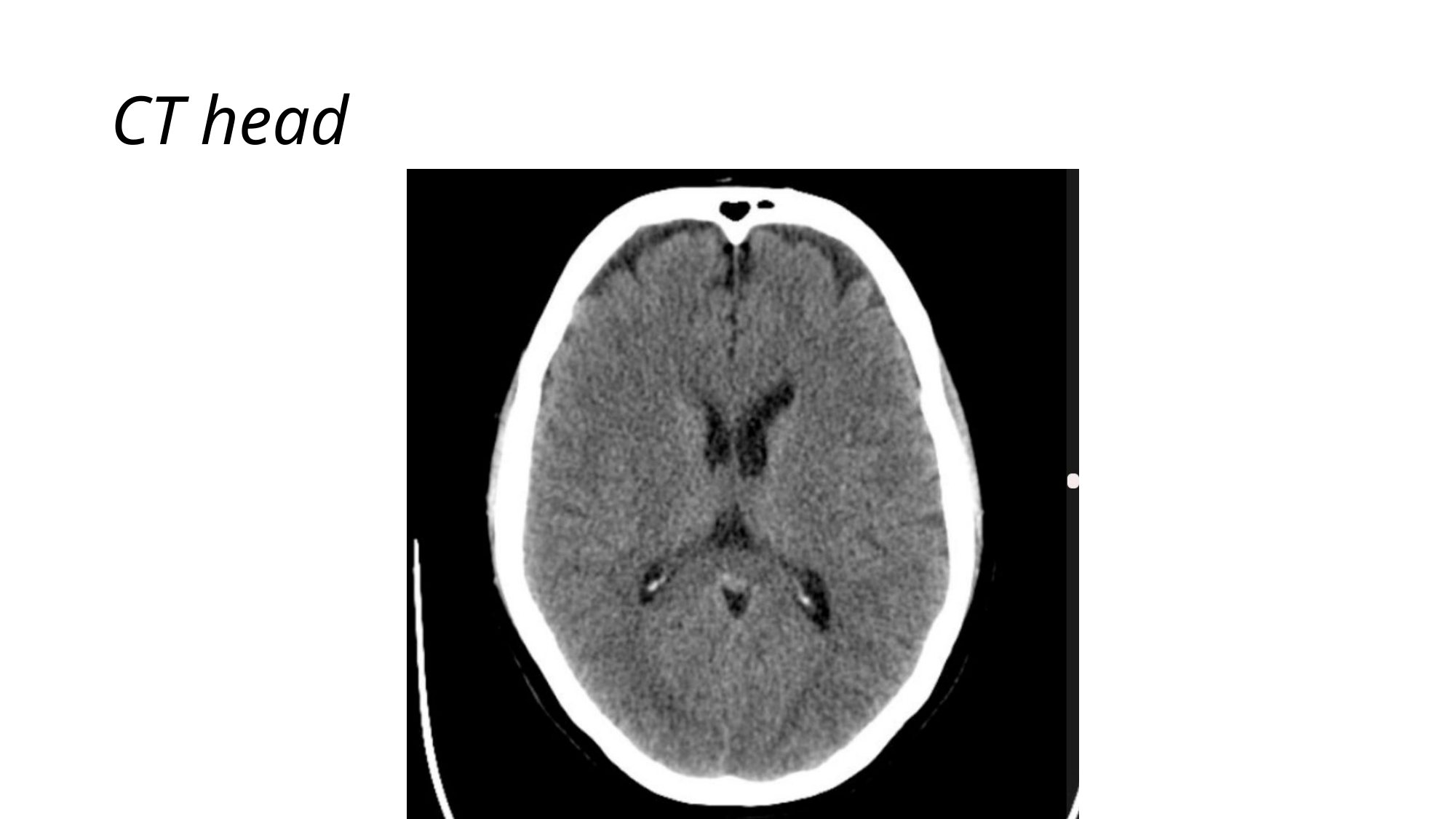

# CT head

## Slide 10
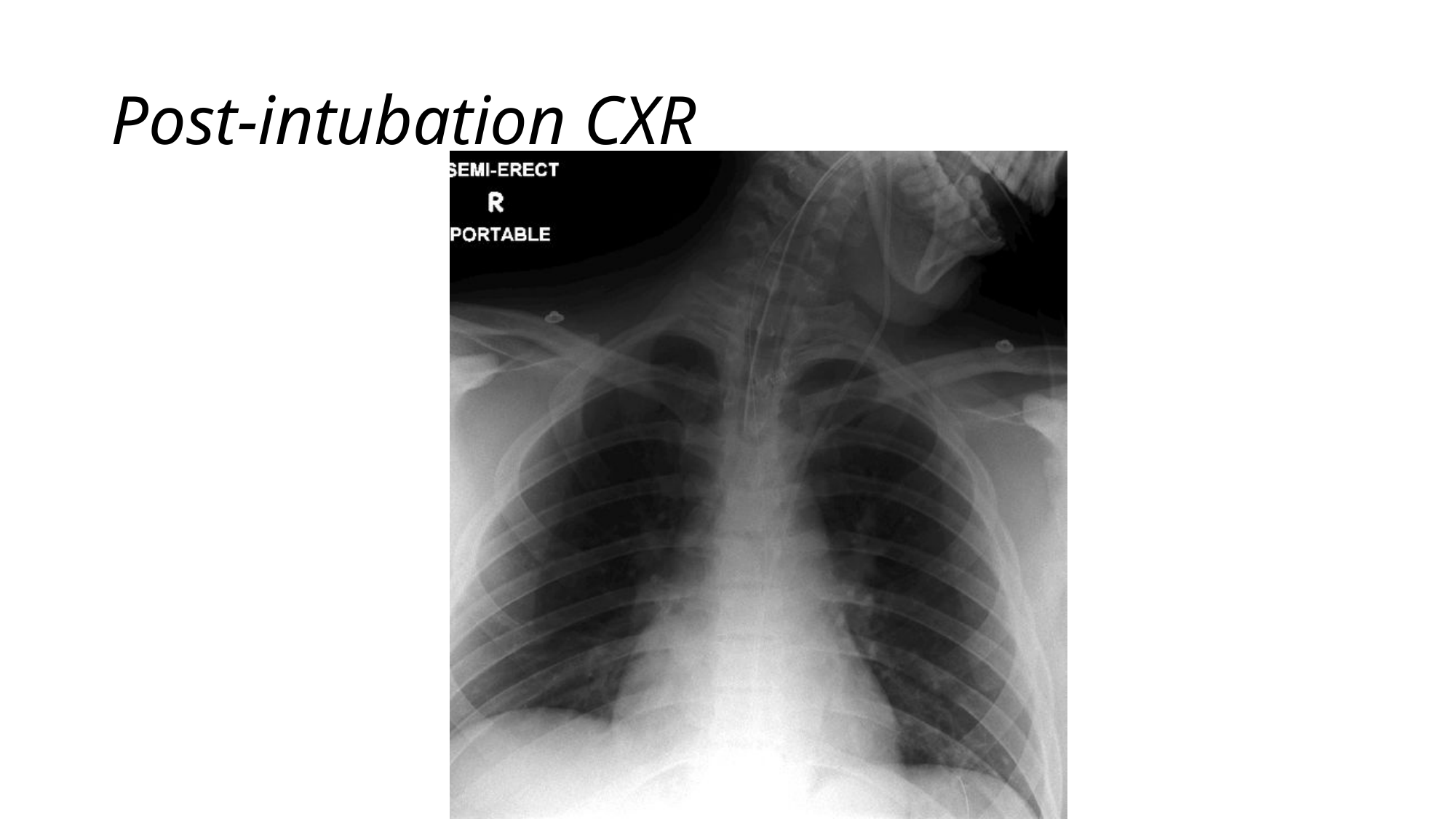

# Post-intubation CXR

## Slide 11
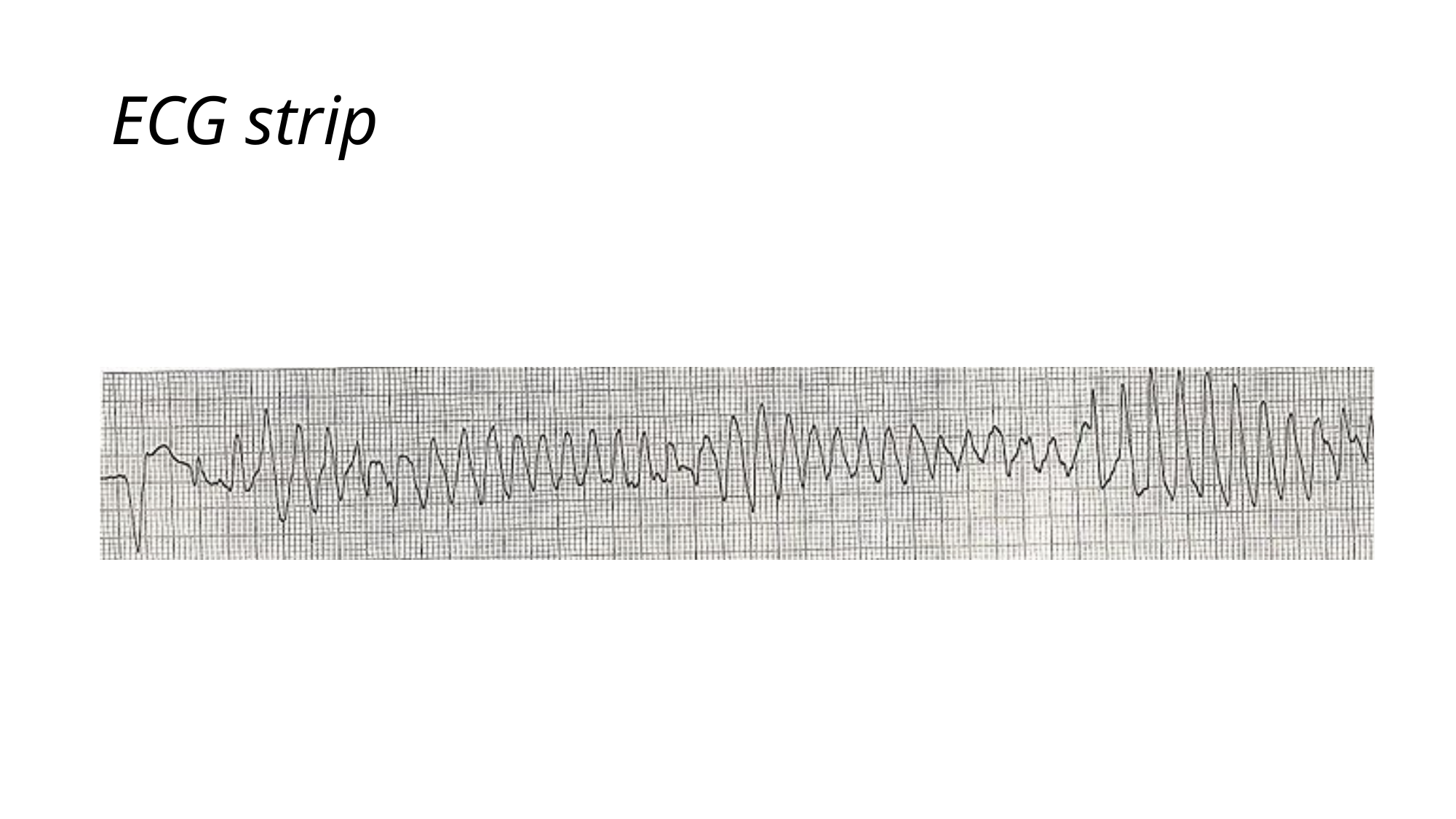

# ECG strip

## Slide 12
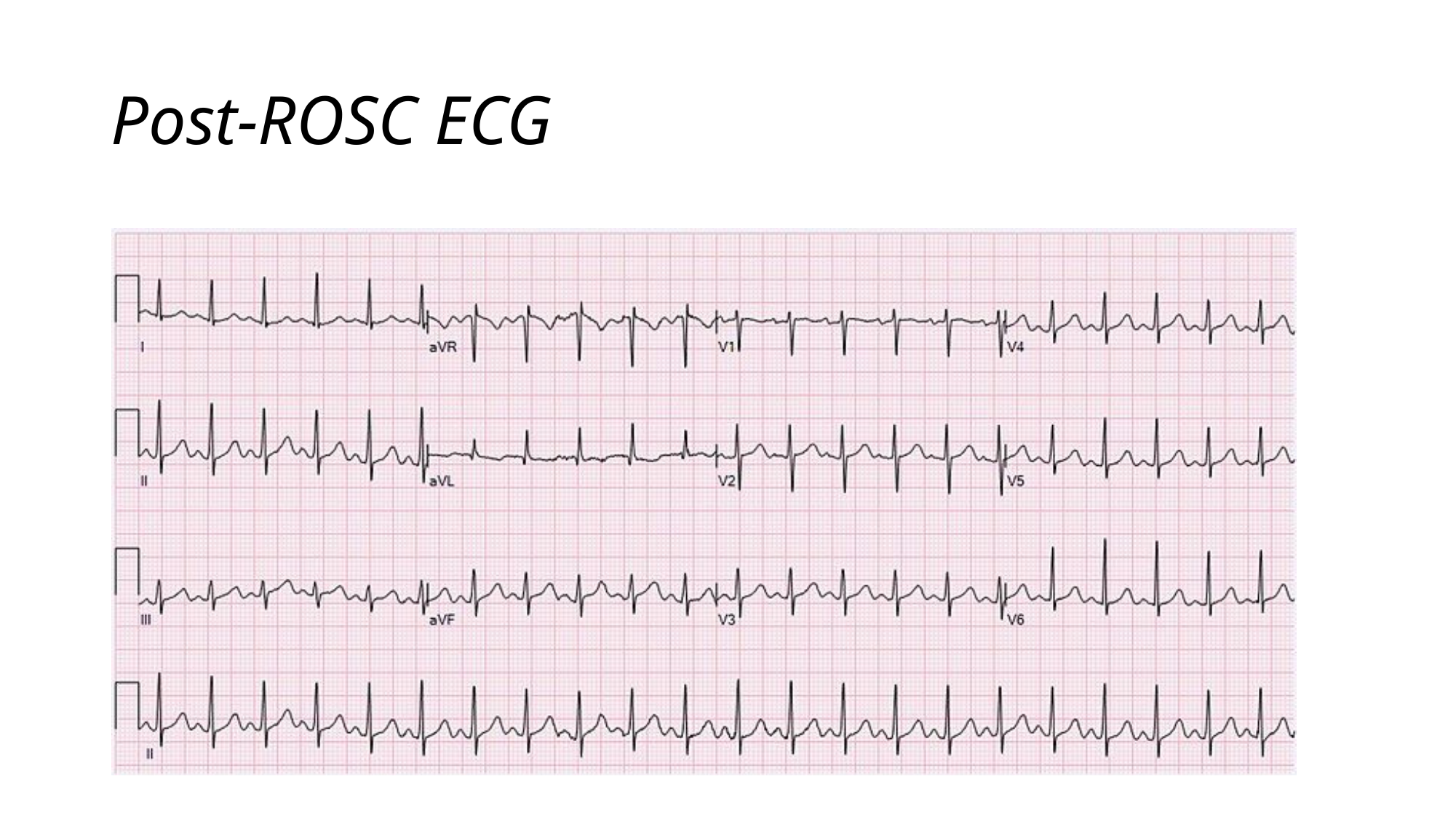

# Post-ROSC ECG
