# Supplementary figures and images for: Torsade de Pointes Due to Hypokalemia and Hypomagnesemia
Source: J Educ Teach Emerg Med. 2022 Oct 15;7(4):S27–51. doi: 10.21980/J8JP8G (PMC10332665; doi:10.21980/J8JP8G)

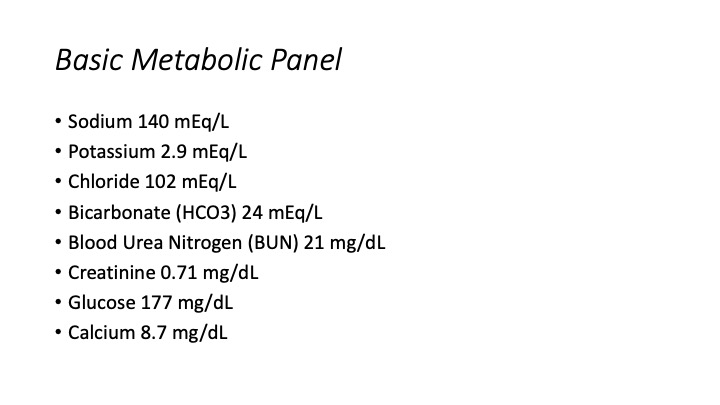

Supplement: Supplementary file 2 [file JETem-7-4-S27-supp2.jpeg]

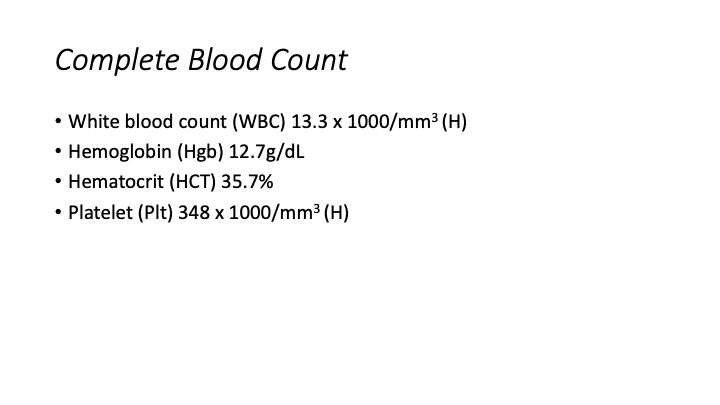

Supplement: Supplementary file 3 [file JETem-7-4-S27-supp3.jpeg]

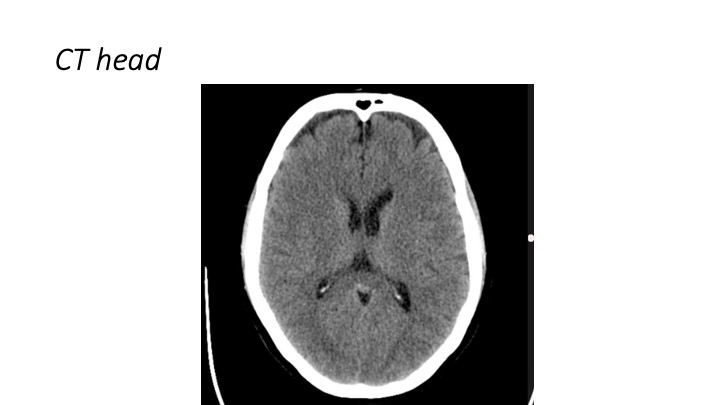

Supplement: Supplementary file 4 [file JETem-7-4-S27-supp4.jpeg]

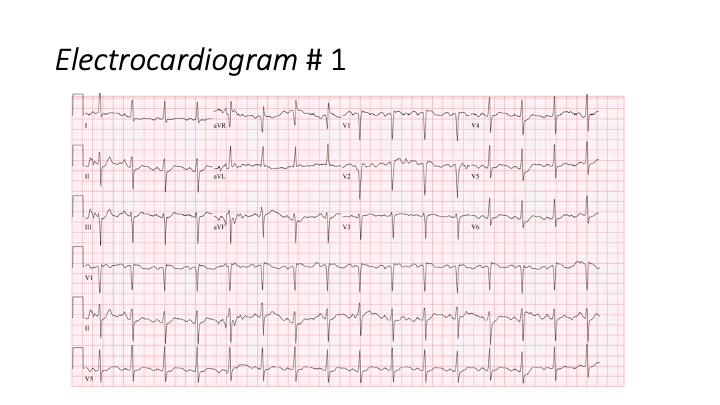

Supplement: Supplementary file 5 [file JETem-7-4-S27-supp5.jpeg]

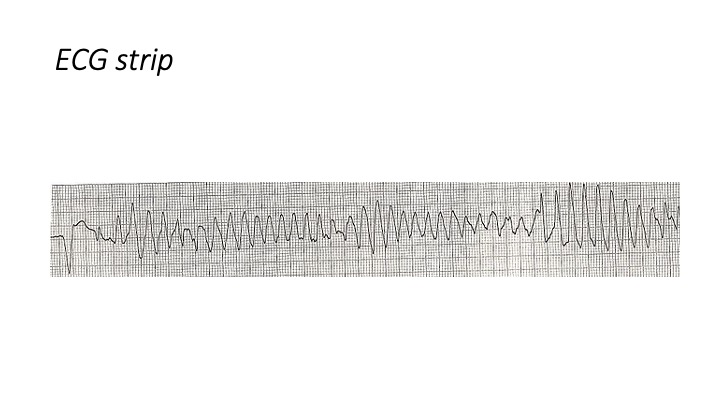

Supplement: Supplementary file 6 [file JETem-7-4-S27-supp6.jpeg]

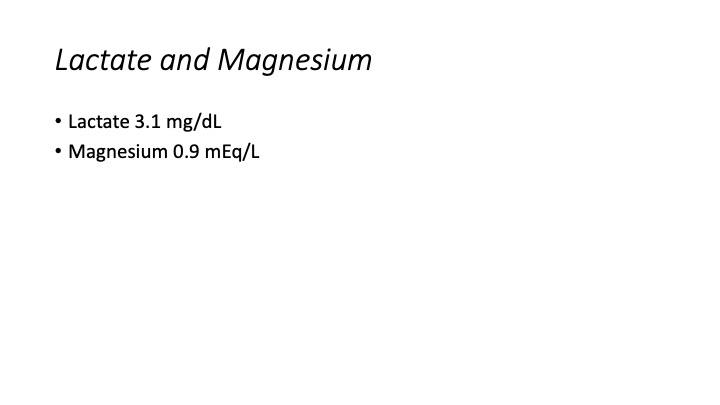

Supplement: Supplementary file 7 [file JETem-7-4-S27-supp7.jpeg]

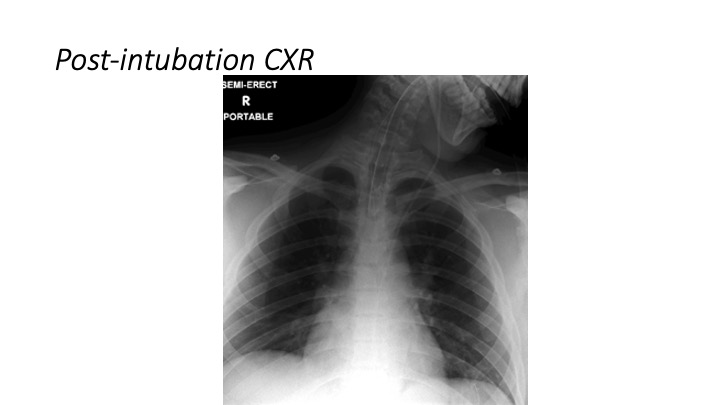

Supplement: Supplementary file 8 [file JETem-7-4-S27-supp8.jpeg]

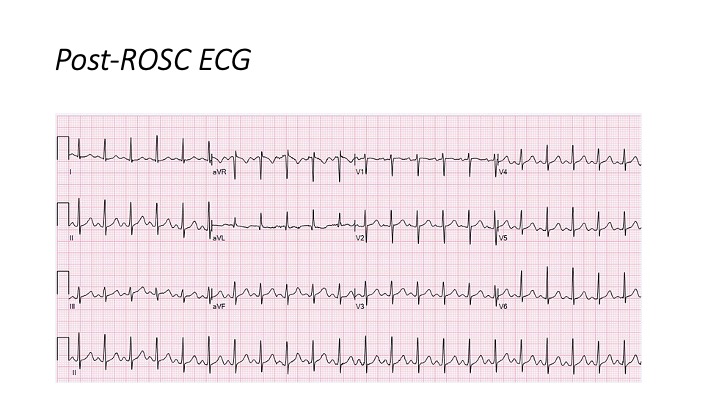

Supplement: Supplementary file 9 [file JETem-7-4-S27-supp9.jpeg]

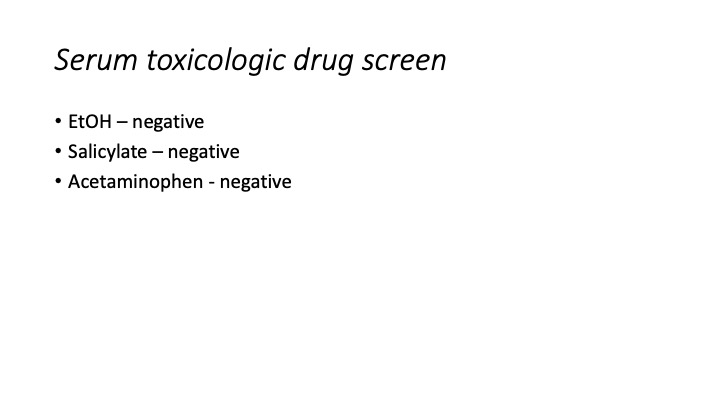

Supplement: Supplementary file 10 [file JETem-7-4-S27-supp10.jpeg]

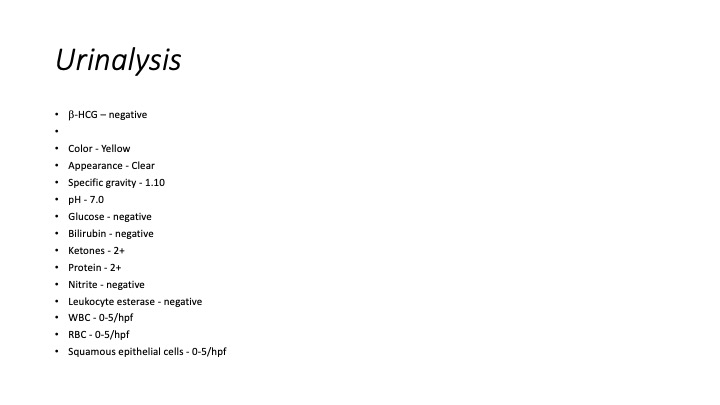

Supplement: Supplementary file 11 [file JETem-7-4-S27-supp11.jpeg]

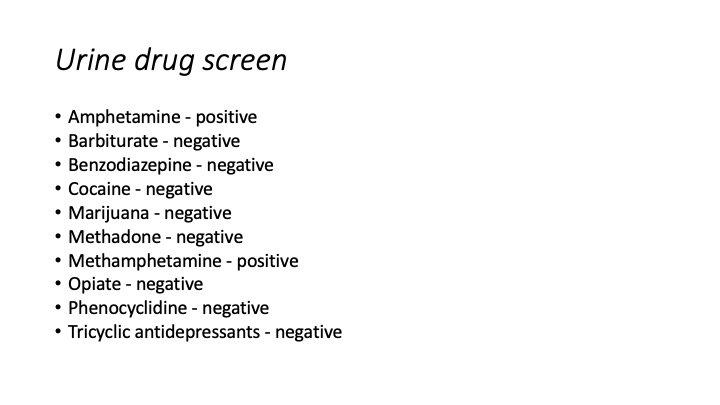

Supplement: Supplementary file 12 [file JETem-7-4-S27-supp12.jpeg]

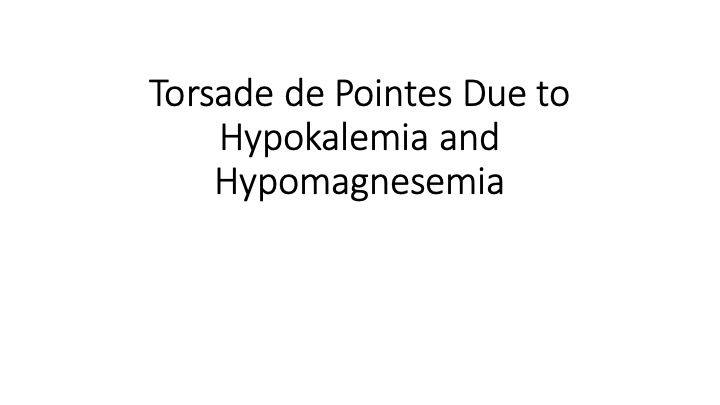

Supplement: Supplementary file 13 [file JETem-7-4-S27-supp13.jpeg]
